# Spike Optimization to Improve Properties of Ferroelectric Tunnel Junction Synaptic Devices for Neuromorphic Computing System Applications

**DOI:** 10.3390/nano13192704

**Published:** 2023-10-05

**Authors:** Jisu Byun, Wonwoo Kho, Hyunjoo Hwang, Yoomi Kang, Minjeong Kang, Taewan Noh, Hoseong Kim, Jimin Lee, Hyo-Bae Kim, Ji-Hoon Ahn, Seung-Eon Ahn

**Affiliations:** 1 Department of IT ∙ Semiconductor Convergence Eng, Tech University of Korea, Siheung 05073, Republic of Korea; bjs3253@tukorea.ac.kr (J.B.); kww0424@tukorea.ac.kr (W.K.); hyunjoo1952@tukorea.ac.kr (H.H.); ahha030@tukorea.ac.kr (Y.K.); dbsk1533@tukorea.ac.kr (M.K.); snrnspdy@tukorea.ac.kr (T.N.); hskim0721@tukorea.ac.kr (H.K.); qlsl000829@tukorea.ac.kr (J.L.); 2 Department of Materials Science and Chemical Engineering, Hanyang University, Ansan 15588, Republic of Korea; hbkim9510@hanyang.ac.kr (H.-B.K.); ajh1820@hanyang.ac.kr (J.-H.A.); 3 Department of Nano & Semiconductor Eng, Tech University of Korea, Siheung 05073, Republic of Korea

**Keywords:** FTJ, synaptic devices, SNN, STDP, neuromorphic computing

## Abstract

The continuous advancement of Artificial Intelligence (AI) technology depends on the efficient processing of unstructured data, encompassing text, speech, and video. Traditional serial computing systems based on the von Neumann architecture, employed in information and communication technology development for decades, are not suitable for the concurrent processing of massive unstructured data tasks with relatively low-level operations. As a result, there arises a pressing need to develop novel parallel computing systems. Recently, there has been a burgeoning interest among developers in emulating the intricate operations of the human brain, which efficiently processes vast datasets with remarkable energy efficiency. This has led to the proposal of neuromorphic computing systems. Of these, Spiking Neural Networks (SNNs), designed to closely resemble the information processing mechanisms of biological neural networks, are subjects of intense research activity. Nevertheless, a comprehensive investigation into the relationship between spike shapes and Spike-Timing-Dependent Plasticity (STDP) to ensure efficient synaptic behavior remains insufficiently explored. In this study, we systematically explore various input spike types to optimize the resistive memory characteristics of Hafnium-based Ferroelectric Tunnel Junction (FTJ) devices. Among the various spike shapes investigated, the square-triangle (RT) spike exhibited good linearity and symmetry, and a wide range of weight values could be realized depending on the offset of the RT spike. These results indicate that the spike shape serves as a crucial indicator in the alteration of synaptic connections, representing the strength of the signals.

## 1. Introduction

Traditional computing systems based on the von Neumann architecture, utilized for information processing over decades, face limitations in handling large-scale unstructured data computations due to their high-power consumption and relatively slow data processing speed in serial operations. To overcome these challenges, recent attention has shifted towards artificial neural networks emulating the functions of the human brain. Particularly, Spike Neural Networks (SNNs) offer advantages in power efficiency and speed by executing operations in parallel through synapses with varying connection strengths, i.e., different weights [1]. Among various learning methods for SNNs, the Spike-Timing-Dependent Plasticity (STDP) learning rule efficiently mimics the information processing mechanisms of biological neural networks by updating synaptic weights based on the temporal correlation of pre- and post-spikes, the electrical signals applied to synapses [2]. Alongside STDP research, there has been a growing interest in small artificial synapse devices to emulate the intricate neural network architecture of the human brain, which typically contains thousands of synapses per neuron. Notably, the idea of using a 2-terminal nanoscale resistive switching device, akin to a memristor, to mimic synapses was introduced by G. S. Snider in 2007 [3]. Subsequently, a variety of 2-terminal memristor devices based on magnetic materials, phase change materials, ferroelectric materials, and transition metals have been extensively studied for synapse application [4,5,6,7,8]. Ferroelectrics, in particular, have been studied for a long time in materials based on perovskite structures, but due to their incompatibility with CMOS processes and the critical limit of losing ferroelectricity at a thickness of a few nanometers, many efforts have been made to overcome them. Then, in 2011, ferroelectricity was reported in 10 nm-thick doped HfO_2_, and the study of devices based on hafnia-based ferroelectric tunnel junctions (FTJs) emerged as a promising alternative in the field of non-volatile memory devices [9,10,11]. FTJ devices utilize the phenomenon of tunneling electrical resistance, which depends on the alignment of the internal polarization of the ferroelectric layer, to realize the memory states. In particular, FTJ devices based on polycrystalline HfO_2_ thin films can determine multiple resistance states because the modulation of the asymmetric tunneling barrier by ferroelectric partial polarization is possible [12,13]. The tunneling current can be extracted in a non-destructive method by applying a read voltage sufficiently small enough not to modulate the tunneling barrier [14]. It is promising for artificial synapse devices applied to SNNs because it has energy-efficient characteristics due to the small amount of current driven by tunneling [15]. However, compared to research on STDP learning with FTJ, there is a scarcity of studies exploring the spike conditions, a critical aspect of STDP.

In this study, we evaluated STDP learning under different spike conditions based on a 6 nm thick HZO FTJ device. To identify effective spike shapes, we performed STDP measurements using spikes of various shapes, and the number of weights, linearity of weight update, and symmetry parameters, which are evaluation criteria for synaptic characteristics, were extracted to suggest efficient spike shapes. Additionally, optimized spike conditions were determined by manipulating the pulse offset and width. The optimized spike was applied to 20 repeated STDP measurements, and the repeated data were applied to neural network simulation based on a CrossSim simulator. The neural network is evaluated through the accuracy of pattern recognition, and the handwritten digit dataset provided by the University of California at Irvine (UCI) and the Mixed National Institute of Standards and Technology (MNIST) was used. The high level of accuracy of pattern recognition demonstrated through simulation highlights the importance of spike optimization.

## 2. Results and Discussion

**Figure 1:**
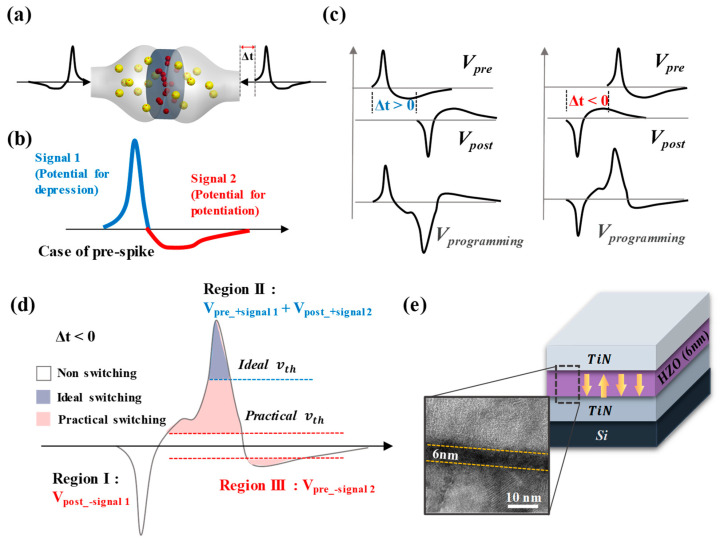
STDP learning mechanism in an artificial synapse device, and analysis of the spike used for STDP. (**a**) The concept of artificial synaptic device mimicking biological STDP learning: interaction of pre- and post-spike. (**b**) The definition of single spike (pre-spike). (**c**) Induction of potentiation and depression by difference of temporal correlation of pre- and post-spikes. (**d**) Schematic representation of a programming spike with overlapping pre- and post-spikes for arbitrary Δt < 0. (**e**) Structure and TEM cross-section image of the TiN/HZO/TiN (metal-ferroelectric-metal (MFM)) FTJ device. The arrow inside the HZO thin film means the direction of polarization.

In STDP learning of an artificial synapse device, the synaptic weight can be regulated by the overlap of voltage signals applied to both ends of the synapse devices (Figure 1a) [16,17,18]. When the spike is applied to the sample, the post-spike applied to the bottom electrode takes on an inverted version of the pre-spike shape. Since the synaptic weight changes according to the temporal correlation of the signals applied to the synapse device, i.e., pre- and post-spike, the condition of the spike applied to the synapse device has a major impact on the STDP learning result [19]. In this case, the pre- and post-spike are signals of the same condition, and the spike used for STDP learning usually has the form of two voltage signals of different polarities in succession, as shown in Figure 1b [20,21,22]. Assuming that a voltage signal of positive polarity is likely to induce depression, a negative voltage signal of opposite polarity is likely to induce potentiation. Since pre- and post-spikes are the same shape but are applied to both ends of the synapse device, the polarity of the pre- and post-spike applied to the synapse is opposite to each other. Assuming that Figure 1b is the voltage signal of the pre-spike, the post-spike is applied in the opposite order to the pre-spike in a signal sequence that is likely to induce potentiation and depression. To facilitate analysis, we define the voltage signal of a pre-spike as a combination of V_pre_+signal 1_ and V_pre_-signal 2_, while the voltage signal of a post-spike received by the synapse is composed of V_post_-signal 1_ and V_post_+signal 2_. Since the pre- and post-spikes are identically shaped spikes, V_pre_+signal 1_ and V_post_-signal 1_ are signals of the same condition with only the polarity reversed. In a biological system, a single spike alone cannot induce a change in synaptic weight. However, when there is a temporal correlation between pre- and post-spike signals, as depicted in Figure 1c, it becomes possible to induce synaptic weight changes, such as potentiation and depression [20]. Potentiation and depression are induced when Δt > 0 and Δt < 0, respectively, where Δt represents the time difference between the pre-spike and post-spike. Δt > 0 indicates that the pre-spike is applied more rapidly than the post-spike, while Δt < 0 indicates the opposite. The induction of potentiation and depression can be easily understood from Figure 1c. When Δt > 0, an overlap of signals occurs between the pre- and post-spike, enhancing the potential to induce potentiation (V_pre_-signal 2_ and V_post_-signal 1_). Consequently, a signal with a higher amplitude is applied compared to a single spike, resulting in potentiation. Conversely, when Δt < 0, the post-spike is applied more rapidly than the pre-spike, leading to an overlap of signals that have the potential to induce depression (V_pre_+signal 1_ and V_post_+signal 2_); in this case, depression can be induced. When Δt has different polarities and the same absolute value, it can be inferred that all conditions, except for the polarity of the overlapped spike, are the same. Additionally, when Δt = 0, the same spike is applied with opposite polarities, resulting in complete cancellation, making it impossible to induce potentiation or depression.

To achieve excellent learning results in STDP, it is essential to analyze the overlapped programming spike formed by two single spikes and optimize the conditions of the single spike. Figure 1d illustrates an arbitrary programming spike when Δt < 0. The programming spike consists of three voltage signals with different polarities presented in succession, and it can be divided into three regions based on the polarity transition, labeled as Region I, II, and III. In an artificial synapse device, the voltage signal amplitude required to induce weight changes is referred to as the threshold voltage (V_th_). In an ideal artificial synapse device, the threshold voltage (ideal V_th_) is set to be equal to or greater than the maximum amplitude of a single spike. Consequently, when a single spike is applied to the device, the weight remains unchanged due to the threshold voltage being higher or equal to the maximum amplitude of the single spike [20]. To induce weight changes, amplification of amplitude must occur due to the temporal correlation between pre- and post-spikes. Consequently, ideally, Region I and Region III, where no amplification of amplitude occurs, do not significantly influence the weight. Therefore, STDP learning based on artificial synapse devices has been studied by applying poling signals with the opposite polarity of Region II before applying the spike, enabling the evaluation of weight change induced by Region II [23,24,25]. However, the threshold voltage of memristor devices, which are under investigation for application as artificial synapse devices, is generally very low [23,26]. Thus, if the threshold voltage of a memristor device is set to the maximum amplitude of a single spike, the amplitude of the programming spike is not amplified enough to induce a resistance state change even when the two spikes are overlapped, and the number of weight states that can be represented is greatly reduced. To address this issue, when pre- and post-spikes overlap and cause amplification, the amplitude of the single spike must be set sufficiently high to induce substantial changes in the memristor’s state. However, when the amplitude is set to a large value, a significant discrepancy arises between the ideal threshold voltage and the practical threshold voltage, which is set equal to the maximum amplitude of a single spike. The blue and red dotted lines in Figure 1d schematically represent, respectively, the ideal and practical threshold voltages. The amplitude of the single spike exceeds the practical threshold voltage, allowing voltage signals in Regions I and III to also surpass the memristor’s threshold voltage, thereby potentially inducing changes in its state. However, as previously mentioned, STDP learning is conducted by applying a poling signal with the opposite polarity to Region II in advance. Consequently, Region I, which has the same polarity as the poling signal, does not affect the state of the memristor. On the other hand, Region III is applied after the memristor’s state has been changed by Region II, thereby potentially influencing the memristor’s state. As a result, it becomes essential to consider both Region II and Region III when configuring the spike condition to facilitate STDP learning effectively. The HZO-based FTJ used in this study is driven by a tunneling mechanism through a 6 nm-thin ferroelectric HZO layer (Figure 1e), making it a suitable device for an artificial synapse device capable of high-density and low current operation. It also has the advantage of having a top-electrode, analog multi-state ferroelectric, bottom-electrode structure that can easily mimic the pre-spike, synaptic weight, and post-spike of biological STDP learning. STDP learning is performed by applying the same spike to the top and bottom electrodes while modulating Δt, and the programming spike applied to the HZO varies accordingly, making it possible to express multiple resistance states.

**Figure 2:**
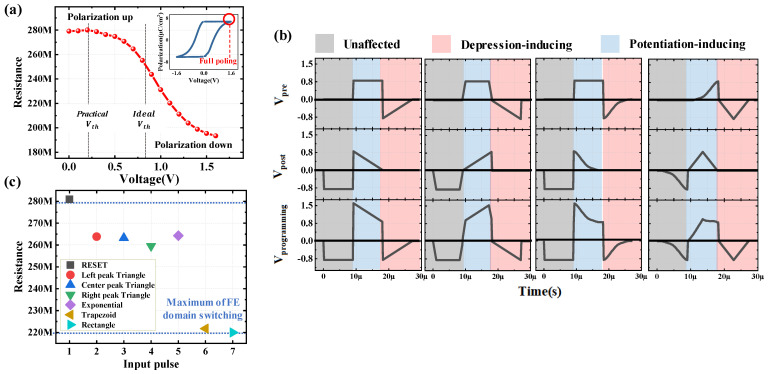
Optimization of single spikes for fabricated HZO-based FTJ devices. (**a**) The resistance state changes with the amplitude of the applied pulse. Inset is the polarization–voltage curve obtained with a PUND measurement. (**b**) Programmed spike of any two combinations of the 6-type pulses, showing the case of Δt (−9 μs), when the reinforcement of the pre- and post-spike is maximized. (**c**) Measuring resistance state change for the 6-type pulses for V_signal 1_ setup.

To define an appropriate amplitude for the single spike in the HZO-based FTJ device, polarization-voltage with a PUND (positive-up, negative-down) measurement and resistance–voltage measurements were conducted. The polarization–voltage curve (Figure 2a inset) obtained by applying a triangle pulse with an amplitude of ±1.6 V shows that all polarizations are switched and saturated at 1.6 V. The measured P–V curve includes only ferroelectric switching contribution that excluded non-ferroelectric switching components from the leakage and dielectric contribution. It also shows that remnant polarization (2Pr) is 12.6 μC/cm^2^, coercive voltages are −0.22 and 0.56 V, and imprint voltage is 0.17 V.

Figure 2a illustrates the change in the resistance state with varying amplitudes of the applied pulse. A poling pulse of negative polarity was applied in advance; the resistance state was measured by sequentially increasing the programming pulse from 0 V to 1.6 V in an interval of 0.1 V. The read voltage was set to 1 V DC bias. Based on these findings, the maximum amplitude capable of inducing transitions from high resistance state (HRS) to low resistance state (LRS) (and vice versa) was set to ±1.6 V. Therefore, to ensure that the maximum state change occurs when both spikes are optimally reinforced and to minimize the range of states that can be modulated by a single spike, the maximum and minimum voltage amplitudes for the single spike were set at an absolute value of 0.8 V. For instance, in the case of the pre-spike, V_pre_+signal 1_ and V_pre_-signal 2_ were set to 0.8 V and −0.8 V, respectively.

To optimize the spike condition, we considered various combinations of two voltage signals, including rectangle, trapezoid, exponential, and three types of triangles (left, center, and right peak). Each pulse condition was set to have a pulse width of 9 μs, as shown in Table 1. To ensure the sufficient charging of the ferroelectric capacitor, the width of signal1 was set longer than the RC time. The programming spike shown in Figure 2b represents a scenario where Δt is −9 μs, leading to maximal reinforcement of both pre- and post-spike and inducing the most significant depression among all spike conditions. In all cases, Regions I, II, and III exhibited negative, positive, and negative polarities, respectively. As previously mentioned, Region I, which has no influence on state changes, was excluded from the analysis. To achieve multi-state capabilities, a large memory window and gradual state changes are required. First, to enlarge the memory window, Region II should be capable of inducing sufficient resistance state changes while minimizing the resistance state changes by Region III. Also, to achieve gradual state changes, the applied signal should be changed gradually. In the case of the STDP learning method, Region II is produced by overlapping V_pre_+signal 1_ and V_post_+signal 2_, which induce depression in the pre- and post-spike, and Region III is produced by V_pre_signal 2_, which induces potentiation in the pre-spike. Therefore, to achieve an ample memory window and gradual state changes, it is advantageous to employ a pulse with a stable amplitude sustained for an appropriate duration in V_pre_+signal 1_. Simultaneously, a pulse with a gradual amplitude change should be applied to V_pre_-signal 2_.

Figure 2c shows the resistance state change measurement for 5-type pulses for the V_signal 1_ setting. Since this is an evaluation of the effect of a single pulse, a pulse under the same conditions as the pulses in Table 1 used for the spikes in Figure 2b, the amplitude was set to 1.6 V for all. The results show that the rectangular pulse induced the most resistance state change from about 280 MΩ to about 220 MΩ, which highlights that the rectangle pulse shape is the most suitable for V_signal 1_. Upon setting V_signal 1_ as a rectangle pulse, it is reasonable to anticipate that V_signal 2_ would require a pulse with a gradual amplitude change, making the triangle pulse a suitable candidate. Consequently, the suitable pulse shape for V_signal 2_ is expected to be the triangle pulse. With V_signal 1_ fixed as a rectangle pulse, we proceeded to conduct STDP measurements by applying three types of triangle pulses (left, center, and right peak) as V_signal 2_. The spike configurations used are as follows: rectangle—left peak triangle (RT_left_); rectangle—center peak triangle (RT_center_); rectangle—right peak triangle (RT_right_). The pulse conditions used in this experiment are identical to those listed in Table 1.

**Figure 3:**
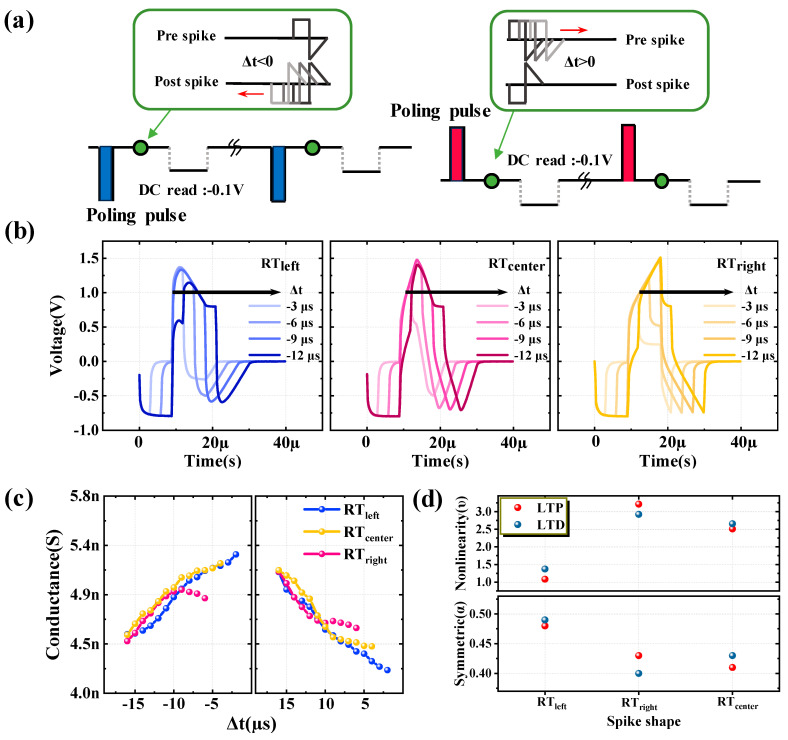
Measuring and analyzing STDP learning for spike shape optimization. (**a**) Measurement sequence of STDP learning with RT_left_ spike. (**b**) For RT_left_, RT_center_, and RT_right_ spikes, the shape of the programming spike is applied to the HZO FTJ. Only the cases with Δt = −3, −6, −9, and −12 μs were plotted. (**c**) STDP measurement results for each spike. LTP curve plotted when Δt > 0; LTD curve plotted when Δt < 0. (**d**) Analyzing linearity and symmetry characteristics of LTD and LTP curves.

Figure 3a illustrates the measurement sequence of STDP learning, with the RT_left_ spike used as an example. The sequence remains identical for all three spike types, proceeding as follows: For cases where Δt decreases in the negative direction from 0, the order of events is poling pulse of negative polarity, pre-spike and post-spike, followed by DC read; the interval of Δt is −1 μs. For cases where Δt increases in the positive direction from 0, the order of events is poling pulse of positive polarity, pre-spike and post-spike, followed by DC read; the interval of Δt is +1 μs. The Δt values are restricted within the range of −18 μs to +18 μs to ensure temporal correlation between pre- and post-spike. During each programming spike, the device’s state modified by the spike is measured as current data under a DC bias of −0.1 V. Regarding the three types of spikes, RT_left_, RT_center_, and RT_right_, the actual programming spike shapes applied to the HZO layer can be observed in Figure 3b. Here, Region I is not considered because it does not affect the state and has the same shape for all three types of spikes. First, for the spike RT_left_, it can be observed that at Δt = −3 μs and −6 μs, Region II inducing potentiation has the highest amplitude, and as Δt increases, the amplitude sequentially decreases. Simultaneously, at Δt = −3 μs, Region III inducing depression has the lowest amplitude, and as Δt increases, the amplitude sequentially increases. Therefore, it is expected that at Δt = −3 μs, the induction of potentiation will be the highest, and a linearly smaller amount of potentiation is induced as Δt increases. Next, when the spike is RT_center_, it can be observed that as Δt increases, the amplitude of Region II is nearly similar to that of RT_left_, while the amplitude of Region III sequentially increases. On the other hand, for the spike RT_right_, as Δt increases, the amplitude of Region II continuously increases, and the amplitude of Region III remains almost similar. Therefore, it is expected that a larger number of states can be obtained in the order of RT_left_, RT_center_, and RT_right_. Figure 3c presents the results of the STDP measurements for each spike condition. The conductance change curve due to potentiation (long-term potentiation, LTP) is plotted when Δt > 0, and the conductance change curve due to depression (long-term depression, LTD) is plotted when Δt < 0. For each spike condition, only data in the region where a linear change in conductance occurs were plotted.

LTP and LTD characteristics were analyzed within the same conductance range, and it can be seen that RT_left_, RT_center_, and RT_right_ exhibited 14, 13, and 6 distinct states, respectively. Notably, the RT_left_ spike demonstrated the highest number of states, indicating that this particular spike condition induced the most significant diversity in conductance levels. The linearity and symmetric characteristics of weight update (LTD and LTP) in the artificial synaptic device were analyzed using the Symmetric Nonlinearity model.

Linearity and symmetric characteristics are required to achieve high learning accuracy in pattern recognition simulation to evaluate artificial neural networks and were analyzed by fitting the following equations (Equations (1)–(3)) [27]:

(1) $$G=A\times \frac{1}{1+\exp\left[-2v\left(p-\alpha \right)\right]}+B\;$$

where

(2) $$A=\left(G_{max}-G_{min}\right)\times \frac{\exp v+1}{\exp v-1}\;$$

(3) $$B=G_{min}-\frac{\left(G_{max}-G_{min}\right)}{\exp v-1}\;$$

The meaning of each term used in Equations (1)–(3) is as follows: *G_max_* is the maximum conductance in the LTD and LTP characteristic curves; *G_min_* is the minimum conductance; α is a parameter that can evaluate symmetry; and υ is a parameter that can evaluate nonlinearity. α serves as the symmetric center of the LTD and LTP characteristic curves and can take values between 0 and 1. A value closer to 0.5 indicates more symmetric characteristics. On the other hand, υ is used to assess nonlinearity and can take values between 0 and 10, with a value closer to 0 indicating more linear characteristics. The α and υ parameters for each spike can be seen in Figure 3d. Among the three spikes, RT_left_ showed the most outstanding symmetric characteristics with α values of 0.48 for LTP and 0.49 for LTD and the best linearity characteristics with υ of 1.1 and 1.4, respectively. Considering the number of states, symmetric, and linearity characteristics together, it is evident that RT_left_ is the most suitable spike shape for the artificial synaptic device.

**Figure 4:**
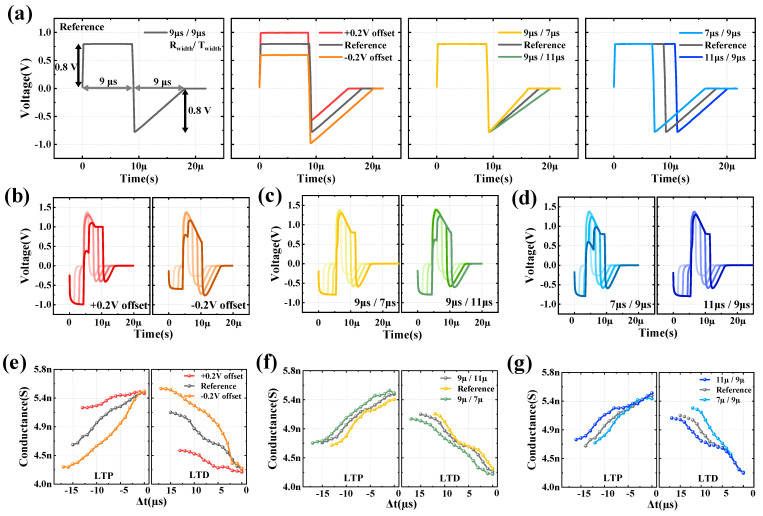
Spike RT_left_ optimization. (**a**) For Spike RT_left_ optimization, establish a spike condition with a modulated offset, as well as the widths of the R pulse and T_left_ pulse. The shape of the programming spike for spike RT_left_ can be characterized by several parameters, including (**b**) Spike shape depending on the level at the offset. +0.2 V offset (left, red line) and −0.2 V offset (right, orange line). (**c**) Spike shape as a function of T_left_ pulse width of 7 μs and 11 μs. (**d**) Spike shape as a function of R pulse width of of 7 μs and 11 μs. Only the cases with Δt = 3, 6, 9, and 12 μs were plotted. STDP measurement results for each spike condition: (**e**) corresponds to (b), (**f**) corresponds to (**c**), and (**g**) corresponds to (**d**).

To optimize the RT_left_ spike, the STDP learning was conducted by varying the spike’s offset, R pulse width, and T_left_ pulse width. As shown in Figure 4a (first), the conditions of the reference spike were set with square and triangular waveforms having peak values of 0.75 V and pulse widths of 9 μs each. The spike modulation conditions were as follows: the offset of the spike was adjusted to −0.2, 0, and +0.2 V (Figure 4a (second)); the R pulse width was set to 7, 9, and 11 μs (Figure 4a (third)); and the T pulse width was set to 7, 9, and 11 μs (Figure 4a (fourth)). In contrast, the amplitude in Region III undergoes a considerable variation, extending to a larger range of values. Conversely, when the offset is +0.2 V, the trends observed are opposite to those seen when the offset is −0.2 V.

In this case, the amplitude in Region I, which does not influence the state, increases, while the amplitude in Region II shows no significant variation. Additionally, the amplitude in Region III undergoes a smaller range of change. The offset modulation case showed the most dramatic applied programming pulse shape change, while the pulse width modulation case showed no significant amplitude change in each region, as shown in Figure 4c,d. The measurement results for Figure 4b–d are plotted in Figure 4e–g and Table 2. As expected, the most dramatic changes were seen when the offset was modulated, with the offset of −0.2 V having the highest number of states of 18. In addition, when the linearity and symmetry characteristics were checked in the same way as in Figure 3c, the case with offset −0.2 V showed the most symmetrical and linear characteristics with α = 0.48 and υ = 1. Therefore, considering the number of states, symmetry, and linearity characteristics, the condition with R width of 9 μs, T width of 9 μs, and offset −0.2 V has the best synaptic characteristics. The performance indicators of synapses are dependent on the shape of the spike. Linearity and symmetric play a role in the performance of neural networks. When data exhibits good linearity and symmetry, the computational process reduces distortion and has a positive impact on accuracy [28]. In Figure 4b, the programming spike shapes for each spike condition are presented. It is essential to pay attention to the results obtained when varying the spike’s offset, particularly when the offset is −0.2 V. The amplitude in Region I, which is known not to influence the device state, decreases, while the amplitude in Region II remains relatively unaffected.

**Figure 5:**
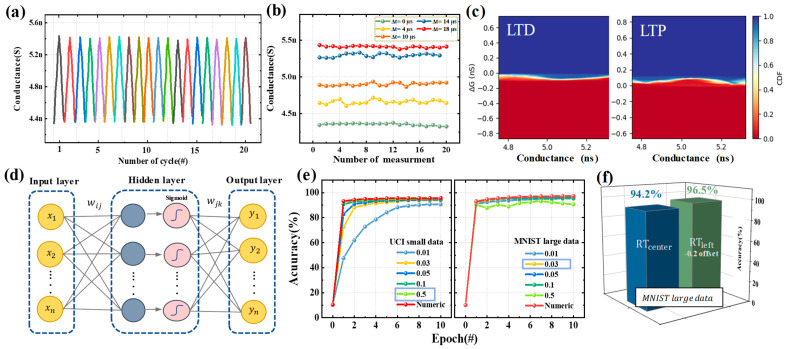
Synaptic characteristics using an optimized spike condition of 6 nm HZO FTJ device. (**a**) Twenty cycles of LTP and LTD with optimized spike conditions. Each color represents an individually measured sequence (**b**) Multi-state reproducibility reproduced by optimized spikes. (**c**) Conductance deviation calculated using cumulative distribution function (CDF) in LTD and LTP. White indicate the probability of each resistance state transitioning to the next resistance state. (**d**) Schematic representation of artificial neural network used in the simulation. (**e**) Pattern recognition accuracy for small and large image datasets as a function of learning rate. (**f**) Accuracy dependence by spike optimization.

Based on the optimized spike conditions, the obtained data was used to perform artificial neural network simulations using the CrossSim simulator provided by Sandia National Laboratories [28,29,30]. The CrossSim simulator is composed of a neural core that converts the input measurement data into pixel brightness and a digital core that computes the weights. The simulation utilized the measurement data obtained from 20 repeated STDP learning experiments with the optimized spike conditions. (Figure 5a). The results of the repeated STDP training experiments show that the resistance level according to the Δt value of the optimized spike is stable. Figure 5b shows the resistance state at five different Δt values (0 µ, 4 µ, 10 µ, 14 µ, and 18 µ) in 20 repeated STDP experiments, and the same resistance state is implemented in the repeated experiments. This allows pattern recognition simulations to be performed on handwritten digit datasets provided by UCI and MNIST using the CrossSim simulator. However, due to the difference between the range of weights obtained from measurements and the range of conductance stored in the crossbar, the measured data needs to be transformed into a format called the Look-up table to be applied to the CrossSim simulator [31]. The Look-up table represents the ΔG-G data, which indicates the conductance state change (ΔG) that occurs when an additional signal is applied to the current device’s conductance state. This table takes into account various non-ideal factors, such as write noise, read noise, and nonlinearity. In Figure 5c, the cumulative distribution function (CDF) represents the updated characteristics of complex conductance states, which includes non-ideal features reflected through the Look-up table. Figure 5d is a schematic of an artificial neural network consisting of an input layer, a hidden layer, and an output layer, and the circled parts of the network are called neurons or nodes [32]. Learning in a neural network refers to the process or algorithm of modifying the weights of neurons that constitute the network [33]. The learning process follows the following steps:

① The pixel brightness values of the UCI and MNIST handwritten digit datasets are input into input layerl; ② the pixel brightness is multiplied by the conductance (G) obtained from the Look-up table, resulting in a summation of values according to Kirchhoff’s current law, which is then output to the hidden layer; ③ the value input to the hidden layer is multiplied by the conductance (G), and the summed value is output to the output layer according to Kirchhoff’s current law; ④ the output value is compared with the known image label (the correct answer), and the cost (error) between them is calculated; and ⑤ the internal weights are adjusted in the direction that reduces the cost, aiming to minimize the error during the learning process.

The handwritten digit datasets used in this study are sourced from UCI and MNIST. The UCI small dataset consists of 8 × 8-pixel handwritten digits and includes 64 nodes, 3823 training samples, and 1797 test samples. On the other hand, the MNIST large dataset consists of 28 × 28-pixel handwritten digits and comprises 784 nodes, 60,000 training samples, and 10,000 test samples. During the training process, each image in the training dataset is used for one epoch, which is considered as one complete iteration of learning.

The accuracy of the neural network at a specific epoch is measured using the test dataset, which is not involved in the learning process and is used solely for evaluation purposes. Before evaluating the pattern recognition accuracy of the artificial neural network based on STDP learning with the optimized spike condition for HZO-based FTJ, the learning rate was optimized. The learning rate plays a crucial role in the learning process, as it determines the step size of weight updates during training. If the learning rate is too small, it may lead to slow learning and hinder the network’s performance, or it might get stuck in local minima without reaching the optimal cost [34]. On the other hand, if the learning rate is too large, it may cause overshooting, where the cost increases instead of converging to the minimum. Therefore, to determine a suitable learning rate for both the UCI small dataset and the MNIST large dataset, we evaluated accuracy by training for up to 10 epochs with learning rates ranging from 0.01 to 0.5, as shown in Figure 5e. A learning rate of 0.5 for the UCI small dataset and 0.03 for the MNIST large dataset exhibited excellent pattern recognition accuracy of 95.4% and 96.5%, respectively. The red line in the graph represents the ideal data accuracy.

These high levels of pattern recognition accuracy can be attributed to the excellent synaptic characteristics achieved through successful spike condition optimization. This is also confirmed by the accuracy comparison based on data obtained by applying optimized and non-optimized spikes, as shown in Figure 5f. At a learning rate of 0.03 on the MNIST dataset, the RT_center_ spike condition demonstrated an accuracy of 94.2%, while the RT_left_ −0.2 V offset spike condition showed an accuracy of 96.5%. This suggests that optimized spikes can further enhance the potential application of synaptic devices in neuromorphic computing systems.

## 3. Conclusions

This study focused on optimizing the spike condition, a key element in STDP learning, using a 6 nm HZO-based FTJ device to improve synaptic characteristics. Through simulations, the research demonstrated the potential application of this device as an artificial synaptic device for neuromorphic computing. The evaluation of synaptic characteristics was based on the number of states, linearity, and symmetry criteria, which were analyzed using STDP learning with various spike conditions. The accuracy of the CrossSim-based artificial neural network was evaluated by repeating the STDP measurement based on the RT_left_ spike with an optimized R width of 9 μs, T width of 9 μs, and offset of −0.2 V 20 times. High pattern recognition accuracy of 95.4% for the UCI dataset and 96.5% for the MNIST dataset was achieved.

In conclusion, this study highlights the importance of optimizing the driving conditions for various artificial synaptic device candidates to effectively utilize their characteristics for neuromorphic computing. Understanding the driving conditions is essential for device design and reproducibility in the context of neuromorphic computing research.

## 4. Experimental

Fabrication: HfZrO_2_ ferroelectric thin films with a thickness of 6 nm were grown on a TiN/SiO_2_/Si substrate by thermal-ALD at 300 °C. Cocktail precursors of cyclopentadienyl-tris(dimethylamino)-hafnium (Hf[Cp(NMe_2_)_3_]) and cyclopentadienyl-tris(dimethylamino)-zirconium (Zr[Cp(NMe_2_)_3_]) were employed in a molar ratio of 0.35:0.65, and ozone was used as reactant gas. The top TiN electrode was deposited by RF magnetron sputtering in an Ar and N_2_ atmosphere with a circular-patterned hard mask (r = 100 µm). Subsequently, the initial amorphous HfZrO_2_ thin films were crystallized in an N_2_ atmosphere at 600 °C for 40 s to stabilize the ferroelectric phase.

Electrical Measurements: Electrical measurements were performed using a parameter analyzer (4200A-SCS, Keithley, USA) with a 4225-PMU. The spike signal was applied to the top and bottom electrodes of the FTJ device. The pulse signal was applied to the top electrode of the FTJ device, and the bottom electrode was grounded. All measurements were performed at room temperature and were preceded by 20,000 field cycles to rule out the wake-up effect in the pristine state.

Neural Network Simulations: The performance of an artificial neural network based on back propagation was simulated using the open-source software CrossSim (Crossbar Simulator, Version 0.2) written in Python provided by Sandia National Laboratories. In the weight update model, the possible weight values of the device were determined by referring to the look-up table created using experimental values rather than virtual simulations.

## Figures and Tables

**Table 1:** Condition of the 6-type pulses.

Pulse Shape	Rising/Falling Time	Pulse Width/Total Pulse Width
Rectangle	200 ns	8.6 μs/9 μs
Trapezoid	1 μs	7 μs/9 μs
Left peak triangle	200 ns (rising), 8.8 μs (falling)	9 μs
Center peak triangle	4.5 μs (rising), 4.5 μs (falling)	9 μs
Right peak triangle	8.8 μs (rising), 200 ns (falling)	9 μs
Exponential	200 ns (rising), 8.8 μs (falling)	9 μs

**Table 2:** Linearity and symmetry values at various spike conditions.

	RT_left_	RT_center_	RT_right_	RT_left_					
				−0.2 V Offset	+0.2 V Offset	7 µs/9 µs	11 µs/9 µs	9 µs/7 µs	9 µs/11 µs
υ	1.12	2.5	3.2	1	1.65	1.91	3.05	1.87	2.7
α	0.48	0.41	0.43	0.48	0.435	0.45	0.41	0.46	0.4

## Data Availability

The data presented in this study are contained within the article.

## References

[1] Kim M.-K., Park Y., Kim I.-J., Lee J.-S. (2020). Emerging materials for neuromorphic devices and systems. Iscience.

[2] Sjöström J., Gerstner W. (2010). Spike-timing dependent plasticity. Spike-Timing Depend. Plast..

[3] Snider G.S. (2007). Self-organized computation with unreliable, memristive nanodevices. Nanotechnology.

[4] Moon K., Lim S., Park J., Sung C., Oh S., Woo J., Lee J., Hwang H. (2019). RRAM-based synapse devices for neuromorphic systems. Faraday Discuss..

[5] Hong X., Loy D.J., Dananjaya P.A., Tan F., Ng C., Lew W. (2018). Oxide-based RRAM materials for neuromorphic computing. J. Mater. Sci..

[6] Lv W., Cai J., Tu H., Zhang L., Li R., Yuan Z., Finocchio G., Li S., Sun X., Bian L. (2022). Stochastic artificial synapses based on nanoscale magnetic tunnel junction for neuromorphic applications. Appl. Phys. Lett..

[7] Nandakumar S., Le Gallo M., Boybat I., Rajendran B., Sebastian A., Eleftheriou E. (2018). A phase-change memory model for neuromorphic computing. J. Appl. Phys..

[8] Nandakumar S., Boybat I., Le Gallo M., Eleftheriou E., Sebastian A., Rajendran B. (2020). Experimental demonstration of supervised learning in spiking neural networks with phase-change memory synapses. Sci. Rep..

[9] Böscke T., Müller J., Bräuhaus D., Schröder U., Böttger U. (2011). Ferroelectricity in hafnium oxide thin films. Appl. Phys. Lett..

[10] Luo Q., Cheng Y., Yang J., Cao R., Ma H., Yang Y., Huang R., Wei W., Zheng Y., Gong T. (2020). A highly CMOS compatible hafnia-based ferroelectric diode. Nat. Commun..

[11] Sulzbach M.C., Estandía S., Gàzquez J., Sánchez F., Fina I., Fontcuberta J. (2020). Blocking of Conducting Channels Widens Window for Ferroelectric Resistive Switching in Interface-Engineered Hf0.5Zr0.5O2 Tunnel Devices. Adv. Funct. Mater..

[12] Kho W., Hwang H., Kim J., Park G., Ahn S.-E. (2023). Improvement of Resistance Change Memory Characteristics in Ferroelectric and Antiferroelectric (like) Parallel Structures. Nanomaterials.

[13] Boyn S. (2016). Ferroelectric Tunnel Junctions: Memristors for Neuromorphic Computing. Ph.D. Dissertation.

[14] Max B., Mikolajick T., Hoffmann M., Slesazeck S. Retention characteristics of Hf 0.5 Zr 0.5 O 2-based ferroelectric tunnel junctions. Proceedings of the 2019 IEEE 11th International Memory Workshop (IMW).

[15] Wu T.-Y., Huang H.-H., Chu Y.-H., Chang C.-C., Wu M.-H., Hsu C.-H., Wu C.-T., Wu M.-C., Wu W.-W., Chang T.-S. Sub-nA low-current HZO ferroelectric tunnel junction for high-performance and accurate deep learning acceleration. Proceedings of the 2019 IEEE International Electron Devices Meeting (IEDM).

[16] Lashkare S., Panwar N., Kumbhare P., Das B., Ganguly U. (2017). PCMO-based RRAM and NPN bipolar selector as synapse for energy efficient STDP. IEEE Electron Device Lett..

[17] Elhamdaoui M., Rziga F.O., Mbarek K., Besbes K. (2022). Spike-time-dependent plasticity rule in memristor models for circuit design. J. Comput. Electron..

[18] Ryu H., Wu H., Rao F., Zhu W. (2019). Ferroelectric tunneling junctions based on aluminum oxide/zirconium-doped hafnium oxide for neuromorphic computing. Sci. Rep..

[19] Kho W., Park G., Kim J., Hwang H., Byun J., Kang Y., Kang M., Ahn S.-E. (2022). Synaptic Characteristic of Hafnia-Based Ferroelectric Tunnel Junction Device for Neuromorphic Computing Application. Nanomaterials.

[20] Linares-Barranco B., Serrano-Gotarredona T. (2009). Memristance can explain spike-time-dependent-plasticity in neural synapses. Nat. Preced..

[21] Mittermeier B., Dörfler A., Horoschenkoff A., Katoch R., Schindler C., Ruediger A., Kolhatkar G. (2019). CMOS compatible Hf0. 5Zr0. 5O_2_ ferroelectric tunnel junctions for neuromorphic devices. Adv. Intell. Syst..

[22] Emelyanov A., Nikiruy K., Serenko A., Sitnikov A., Presnyakov M.Y., Rybka R., Sboev A., Rylkov V., Kashkarov P., Kovalchuk M. (2019). Self-adaptive STDP-based learning of a spiking neuron with nanocomposite memristive weights. Nanotechnology.

[23] Majumdar S., Tan H., Qin Q.H., van Dijken S. (2019). Energy-efficient organic ferroelectric tunnel junction memristors for neuromorphic computing. Adv. Electron. Mater..

[24] Cai Y., Zhang J., Yan M., Jiang Y., Jawad H., Tian B., Wang W., Zhan Y., Qin Y., Xiong S. (2022). Molecular ferroelectric/semiconductor interfacial memristors for artificial synapses. Npj Flex. Electron..

[25] Ma C., Luo Z., Huang W., Zhao L., Chen Q., Lin Y., Liu X., Chen Z., Liu C., Sun H. (2020). Sub-nanosecond memristor based on ferroelectric tunnel junction. Nat. Commun..

[26] Wang Z., Rao M., Midya R., Joshi S., Jiang H., Lin P., Song W., Asapu S., Zhuo Y., Li C. (2018). Threshold switching of Ag or Cu in dielectrics: Materials, mechanism, and applications. Adv. Funct. Mater..

[27] Agarwal S., Plimpton S.J., Hughart D.R., Hsia A.H., Richter I., Cox J.A., James C.D., Marinella M.J. Resistive memory device requirements for a neural algorithm accelerator. Proceedings of the 2016 International Joint Conference on Neural Networks (IJCNN).

[28] Marinella M.J., Agarwal S., Hsia A., Richter I., Jacobs-Gedrim R., Niroula J., Plimpton S.J., Ipek E., James C.D. (2018). Multiscale co-design analysis of energy, latency, area, and accuracy of a ReRAM analog neural training accelerator. IEEE J. Emerg. Sel. Top. Circuits Syst..

[29] Yang S.T., Li X.Y., Yu T.L., Wang J., Fang H., Nie F., He B., Zhao L., Lü W.M., Yan S.S. (2022). High-Performance Neuromorphic Computing Based on Ferroelectric Synapses with Excellent Conductance Linearity and Symmetry. Adv. Funct. Mater..

[30] Song S., Ham W., Park G., Kho W., Kim J., Hwang H., Kim H.B., Song H., Ahn J.H., Ahn S.E. (2022). Highly stable artificial synapses based on ferroelectric tunnel junctions for neuromorphic computing applications. Adv. Mater. Technol..

[31] Majumdar S. (2022). An efficient deep neural network accelerator using controlled ferroelectric domain dynamics. Neuromorphic Comput. Eng..

[32] Max B., Hoffmann M., Mulaosmanovic H., Slesazeck S., Mikolajick T. (2020). Hafnia-based double-layer ferroelectric tunnel junctions as artificial synapses for neuromorphic computing. ACS Appl. Electron. Mater..

[33] Zayer F., Dghais W., Benabdeladhim M., Hamdi B. (2019). Low power, ultrafast synaptic plasticity in 1R-ferroelectric tunnel memristive structure for spiking neural networks. AEU-Int. J. Electron. Commun..

[34] Tran-Ngoc H., Khatir S., De Roeck G., Bui-Tien T., Wahab M.A. (2019). An efficient artificial neural network for damage detection in bridges and beam-like structures by improving training parameters using cuckoo search algorithm. Eng. Struct..

